# The Slipper Fracture: Revisited

**DOI:** 10.7759/cureus.38607

**Published:** 2023-05-05

**Authors:** Carolina Alvarez, Joshua Chen, Nick I Pilla, William Hennrikus

**Affiliations:** 1 Orthopaedics, Penn State College of Medicine, Hershey, USA; 2 Orthopaedics, Penn State Health Milton S. Hershey Medical Center, Hershey, USA

**Keywords:** angulation, metaphysis, slipper, fracture, radius

## Abstract

Objectives

The term “slipper fracture” is used to describe a fracture of the radius at the junction of the metaphysis and diaphysis. This fracture has an “evil” reputation because it often angulates in the cast. Historically, there have been differing opinions on the optimal way to cast slipper fractures either with a long arm cast in pronation or a long arm cast in supination to prevent angulation. The purpose of this study is to report the outcomes of “slipper fractures” treated with casting.

Methods

Sixteen slipper fractures were retrospectively reviewed. Electronic medical records (EMRs) and radiographs were analyzed to gather data on body weight, cast type, cast position, cast index, loss of reduction, cast wedging, repeat reduction, surgery, and amount of remodeling.

Results

The average age of the patients was eight years old. The average body weight was 30.4 kg. Initial casting included 14 long arm casts in neutral, one short arm cast, and one sugar tong splint. The average cast index was 0.87. Only one cast had a cast index of less than 0.8. This fracture was treated with a long arm cast and did not displace. Of the fractures, 94% lost reduction in the cast and angulated an average of 26 degrees. Two cases were treated with a cast wedge; 13 were observed. Remodeling occurred at an average rate of 2.7 degrees/month. The average remodeling measured at the last follow-up was 15 degrees.

Conclusion

Slipper fractures are difficult to treat due to the angulation of the fracture in the cast. The current study indicates that a long arm cast, appropriate cast index, and cast position are key to preventing the loss of reduction or angulation of a slipper fracture.

## Introduction

In 1983, Rang coined the term “slipper fracture” for a fracture of the radius at the junction of the metaphysis and diaphysis. According to Rang, this fracture has an “evil” reputation because angulation often occurs after casting [1]. Another author, Andrew Pollen [2], wrote that the tendency for these injuries, without initial clinical deformity, to emerge from their plaster a few weeks later with considerable deformity was alarming. In some cases, these fractures are misdiagnosed as innocent and treated with a splint or short arm cast, which can lead to angulation in the cast and a deformity that may be noticed a few weeks too late to correct [3]. Because the slipper fracture is a supination injury, Rang treated this fracture in a long arm cast in full pronation because, theoretically, pronation inhibits the supination deformity and the periosteum locks the fracture in place [1]. Conversely, Pollen [2] recommended a long arm cast in supination because, in supination, the brachioradialis muscle would theoretically hold the fracture reduction in place. Pollen explained that, although the slipper fracture is a supination injury, pronation places the brachioradialis muscle under tension, which may tilt the fracture into angulation and displacement. Instead, according to Pollen, the supinator muscle should be in a relaxed position, and therefore, the arm should be placed in supination. Collectively, Rang and Pollen also highlighted two important principles when treating a slipper fracture: first, the slipper fracture must be aligned straight and, second, plaster cast immobilization must be meticulous [1,2]. Years later, multiple authors defined meticulous casting using a cast index measurement [4,5]. The cast index is the ratio measured on radiographs of the lateral to anteroposterior diameters of the cast at the fracture site. A cast index of <0.8 demonstrates a meticulously applied cast that will minimize the risk of loss of reduction [5]. The purpose of this study is to report the outcomes of slipper fractures treated with casting. The paper was previously presented at the annual meeting of the American Academy of Orthopaedic Surgeons 2022 and at the annual meeting of the Pennsylvania Orthopaedic Society 2022.

## Materials and methods

Methods

The College of Medicine institutional review board (IRB) approved the study (approval number 17709). This is a retrospective study performed at a large academic rural health system. The treatment of 16 consecutive slipper fractures supervised by the senior author (WH) between 2018 and 2020 was retrospectively reviewed.

Inclusion criteria

Patients less than 18 years of age with nonsurgically treated slipper fractures were included. A slipper fracture was defined as a pediatric distal forearm fracture at the junction of the metaphysis and diaphysis [1]. All fractures were complete and non-displaced.

Electronic medical records (EMRs) and radiographs were abstracted for data, including patient age, patient sex, growth plate status, patient body weight, cast type, cast position, cast index, presence of loss of fracture reduction/angulation, need for cast wedging, need for repeat reduction, need for surgery, remodeling amount, and follow-up. Descriptive statistics were used to summarize the demographics, injury characteristics, incidence of complications, and radiographic outcomes.

## Results

Sixteen slipper fractures were studied. The average patient age was eight years old (range: 3-13). All patients had open growth plates. The study included 10 males and six females. Body weight ranged from 17.1 kg to 49.2 kg, with an average body weight of 30.4 kg. Body mass index (BMI) ranged from 14.3 kg/m^2^ to 27.2 kg/m^2^ with an average of 18.6 kg/m^2^ (Table 1).

**Table 1:** Patient demographics

	Total
Age	
0-5	3
6-10	9
11-13	4
Sex	
Female	6
Male	10
Weight	
Underweight (<5%)	1
Healthy weight (5%-84%)	10
Overweight (85%-94%)	2
Obese (>95%)	3
Dominant arm	
Right	5
Left	3
Unknown	8
Location of Fracture	
Right	6
Left	10
Mechanism of injury	
Fall	4
Bike, roller blades, skateboard	6
Swings	3
Playground	3
Open growth plates	
Yes	16
No	0

**Figure 1:**
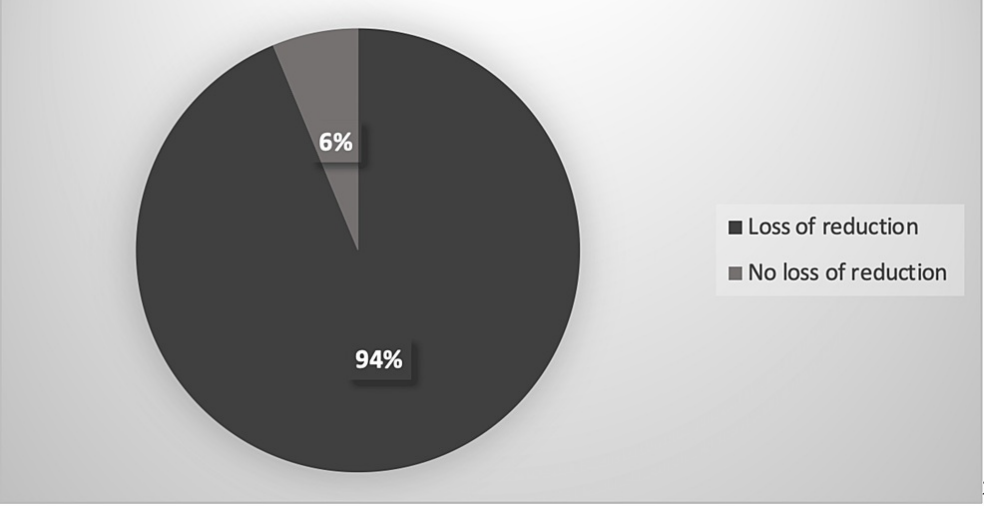
Loss of reduction

Six cases were isolated radial fractures, and 10 were radius and ulna fractures. The fractures were initially treated by a third-year orthopedic resident on call in the emergency department (ED) and supervised by an orthopedic attending via phone as needed. Follow-up was done by third- and fourth-year orthopedic residents supervised by the senior author (WH) one to three weeks later at the pediatric orthopedic fracture clinic. Initial treatment in the ED included 14 treated with a long arm cast in neutral, one treated in a short arm cast, and one treated in a sugar tong splint. Long arm casts were left in place for four weeks with a transition to either a short arm cast or removable wrist splint for two weeks. The short arm cast and sugar tong splint were left in place for four weeks and transitioned to a removable wrist splint for two weeks. The average cast index was 0.87 (range: 0.74-1.04). Fifteen of the 16 fractures (94%) lost reduction in the cast (Figure 1).

**Figure 2:**
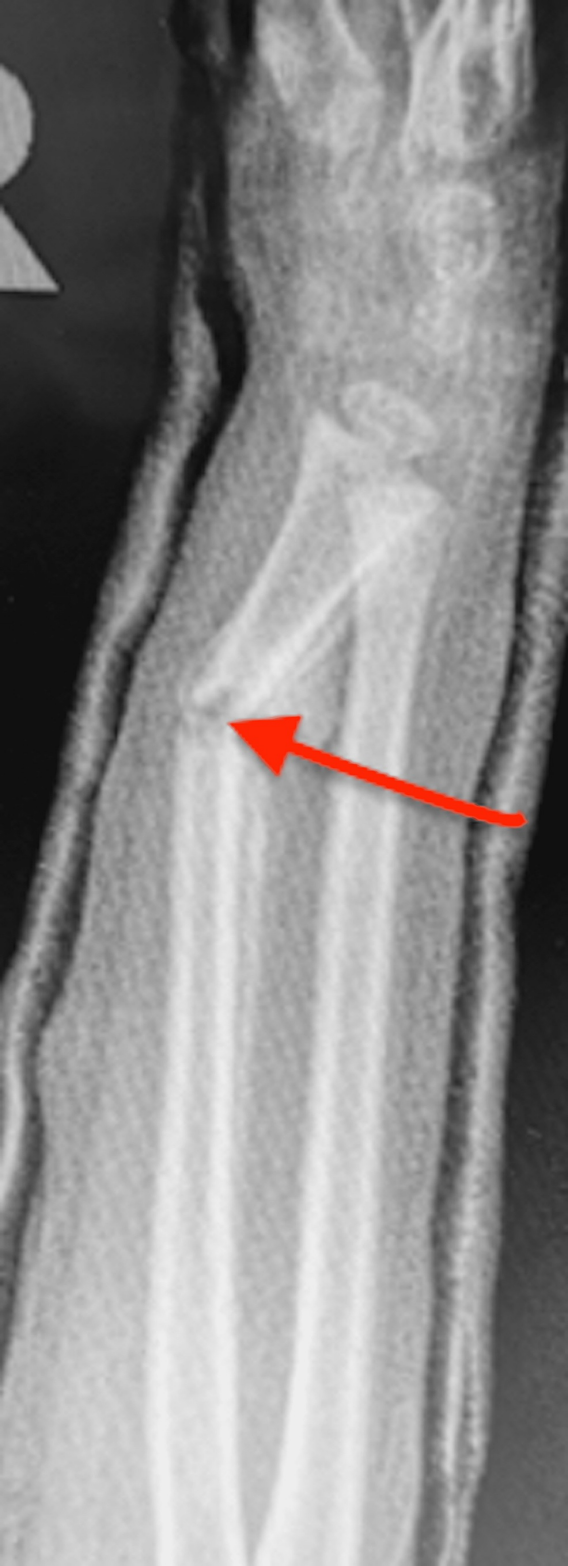
Angulated slipper fracture at four weeks after transition from a long arm to a short arm cast (arrow)

Only one cast had a cast index of <0.8. That fracture was treated in a long arm cast and did not displace. The average angle of lost reduction was 26 degrees (range: 16-41) (Figure 2).

**Figure 3:**
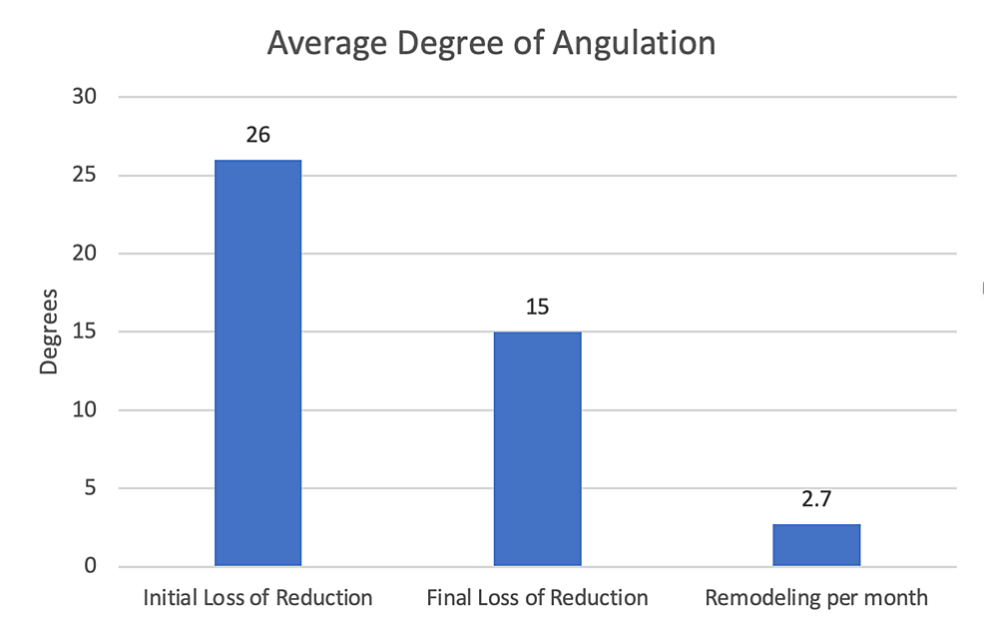
Fracture angulation: average angulation degree due to loss of reduction in the cast, average degree of angulation at the final follow-up, and average degree of remodeling per month

Of the patients who lost reduction, initial treatment had been 13 long arm cast in neutral, one short arm cast, and one sugar tong splint. Two cases that lost reduction were treated with a cast wedge, 14 were observed and followed for remodeling, none were treated with repeat reduction, and none were treated with surgery. In the cases that lost reduction, remodeling occurred at an average of 2.7 degrees per month. Final follow-up averaged five months (range: 1-11). Remodeling at the final follow-up averaged 15 degrees (range: 3-26) (Figure 3).

## Discussion

Distal radius fractures are one of the most common fractures in pediatric patients, often occurring from a simple fall during sports or play activity [6,7]. As Rang wrote in 1983, the slipper fracture is “evil” [1]. The slipper fracture is a non-displaced complete fracture of the distal metaphyseal-diaphyseal junction of the radius or radius and ulna that displaces after initial casting. This fracture has earned a bad reputation because of angulation and resulting deformity that occurs in the cast during treatment [1,2]. However, Rang and Pollen disagreed on how this fracture should be treated to prevent angulation. Rang prefers the arm to be positioned in pronation, while Pollen recommends the arm in supination. While Rang and Pollen disagree on the position of the arm for treatment, they both acknowledge that the fracture must be aligned straight and have meticulous plaster application and molding [1,2]. We failed to carefully read Rang and Pollen’s recommendations about the slipper fracture until researching the literature for the current manuscript. Unfortunately, in the current study, no patient’s slipper fracture was casted in either pronation or supination. Chess et al. [4] and Kamat et al. [5] recommend that one way to determine if a cast was meticulously placed was to measure the cast index. An ideal cast index, or the ratio of the lateral to anteroposterior cast diameters measured on radiographs, is less than 0.8. If the cast index is greater than a 0.8 ratio, the fracture is prone to re-displacement [5]. The current study confirms the utility of the cast index. Fifteen of 16 fractures with a cast index of >0.8 displaced. The one long arm cast with a cast index of 0.74 did not displace. After the data for the current study were analyzed, we performed in-service training for the resident physicians about cast position and molding for this fracture.

In fact, because of the high risk that distal forearm fractures at the junction of the metaphysis and diaphysis will angulate in a cast, some authors recommend more aggressive initial surgical treatment with closed reduction, K-wire fixation, and casting [8,9]. One study demonstrated that when comparing distal radius and distal radius and ulna fractures treated with a long arm cast compared to a K-wire and a long arm cast, those treated without a K-wire sustained more displacements in the cast. However, at a five-year follow-up, there were no differences between the two groups, and all fractures were eventually remodeled [8]. Pollen [2] also reported that young patients with open growth plates are able to correct the angulation deformity over time with fracture remodeling. In the current study, we did not use K-wire fixation. The average age of the patients in the current study was only eight years old. In addition, all displaced fractures were remodeled or were remodeling at an average rate of 2.7 degrees per month at the last follow-up. However, although not studied in the current paper, patients older than age 12 may benefit from initial K-wire fixation due to the lack of remodeling potential at this older age.

Another factor that can play a role in pediatric forearm fracture incidence and healing is obesity. Since 1999, obesity prevalence among children and adolescents has increased from an estimated 13.9%-19.7% in 2020 [10,11]. For example, obesity is associated with an increased risk of falling [12,13]. Some studies have reported that obese children are more likely to sustain an upper extremity fracture than nonobese children from ground-level falls [14-16]. In addition, one study reported that obese children have a higher rate of loss of fracture reduction than that healthy-weight children [17-19]. In the current study, only two of the 16 (12.5%) patients were overweight, and three (18.75%) were obese [20]. The study suggests that weight does not impact the incidence of sustaining a slipper fracture. However, all five fractures in patients who were overweight or obese angulated in the cast.

Lastly, it is important to recognize the difference between a slipper fracture and a buckle fracture. Occasionally, the slipper fracture may be initially mistaken for the more innocent buckle fracture. Buckle, or torus, fractures are compression injuries of one cortex with no risk for angulation or displacement [21,22]. They can be treated with either a splint or short arm cast [23-26]. In contrast, a slipper fracture is an initially non-displaced complete fracture of the distal forearm that displaces in the cast [1,2]. As the current study demonstrates and previous authors have advised, the slipper fracture is best treated with a long arm cast applied with meticulous molding [1,2,5].

This current study has limitations. The sample size is small, cases were collected from only one medical center, and the follow-up was short. No cast was applied in pronation or supination as recommended by Rang and Pollen. Cast molding, as documented by the cast index of >0.8, was inadequate in 15 of 16 cases. We were unable to compare the cast index groups due to a small sample size.

## Conclusions

In conclusion, a long arm cast, appropriate cast index, and cast position are key to preventing the loss of reduction or angulation of a slipper fracture. Close follow-up is warranted. In children older than 12 years of age, K-wire fixation may be indicated. Because the justification to apply the cast in supination or pronation is not strong, we recommend a future prospective study treating slipper fractures with a long arm cast in supination compared to pronation.

## References

[1] Wenger DR, Pring ME, Rang M (2015). Radius and ulna. Rang's children's fractures.

[2] Pollen AG (1973). Fractures and dislocations in children. https://catalog.library.vanderbilt.edu/permalink/01VAN_INST/13em2a7/alma991023435139703276.

[3] Schranz PJ, Fagg PS (1992). Undisplaced fractures of the distal third of the radius in children: an innocent fracture?. Injury.

[4] Chess DG, Hyndman JC, Leahey JL, Brown DC, Sinclair AM (1994). Short arm plaster cast for distal pediatric forearm fractures. J Pediatr Orthop.

[5] Kamat AS, Pierse N, Devane P, Mutimer J, Horne G (2012). Redefining the cast index: the optimum technique to reduce redisplacement in pediatric distal forearm fractures. J Pediatr Orthop.

[6] Nellans KW, Kowalski E, Chung KC (2012). The epidemiology of distal radius fractures. Hand Clin.

[7] Sankar WN, Beck NA, Brewer JM, Baldwin KD, Pretell JA (2011). Isolated distal radial metaphyseal fractures with an intact ulna: risk factors for loss of reduction. J Child Orthop.

[8] Rebello GN (2022). CORR insights®: do we need to stabilize all reduced metaphyseal both-bone forearm fractures in children with K-wires?. Clin Orthop Relat Res.

[9] McLauchlan GJ, Cowan B, Annan IH, Robb JE (2002). Management of completely displaced metaphyseal fractures of the distal radius in children. A prospective, randomised controlled trial. J Bone Joint Surg Br.

[10] Sanyaolu A, Okorie C, Qi X, Locke J, Rehman S (2019). Childhood and adolescent obesity in the United States: a public health concern. Glob Pediatr Health.

[11] (2023). Centers for Disease Control and Prevention: Childhood obesity facts. https://www.cdc.gov/obesity/data/childhood.html.

[12] Corbeil P, Simoneau M, Rancourt D, Tremblay A, Teasdale N (2001). Increased risk for falling associated with obesity: mathematical modeling of postural control. IEEE Trans Neural Syst Rehabil Eng.

[13] Alemdaroğlu KB, Iltar S, Cimen O, Uysal M, Alagöz E, Atlihan D (2008). Risk factors in redisplacement of distal radial fractures in children. J Bone Joint Surg Am.

[14] Davidson PL, Goulding A, Chalmers DJ (2003). Biomechanical analysis of arm fracture in obese boys. J Paediatr Child Health.

[15] Kim SJ, Ahn J, Kim HK, Kim JH (2016). Obese children experience more extremity fractures than nonobese children and are significantly more likely to die from traumatic injuries. Acta Paediatr.

[16] Manning Ryan L, Teach SJ, Searcy K (2015). The association between weight status and pediatric forearm fractures resulting from ground-level falls. Pediatr Emerg Care.

[17] Vescio A, Testa G, Sapienza M (2022). Is obesity a risk factor for loss of reduction in children with distal radius fractures treated conservatively?. Children (Basel).

[18] DeFrancesco CJ, Rogers BH, Shah AS (2018). Obesity increases risk of loss of reduction after casting for diaphyseal fractures of the radius and ulna in children: an observational cohort study. J Orthop Trauma.

[19] Liu Y, Liu C, Guo D, Wang N, Zhao Y, Li D (2021). Effect of childhood overweight on distal metaphyseal radius fractures treated by closed reduction. J Orthop Surg Res.

[20] (2023). Centers for Disease Control and Prevention: CDC extended BMI-for-age growth charts. https://www.cdc.gov/growthcharts/Extended-BMI-Charts.html.

[21] Dua K, Stein MK, O'Hara NN (2019). Variation among pediatric orthopaedic surgeons when diagnosing and treating pediatric and adolescent distal radius fractures. J Pediatr Orthop.

[22] MacNeille R, Hennrikus WL (2018). Value-based treatment of common pediatric fractures by primary care. Clin Pediatr (Phila).

[23] Boutis K, Willan A, Babyn P, Goeree R, Howard A (2010). Cast versus splint in children with minimally angulated fractures of the distal radius: a randomized controlled trial. CMAJ.

[24] Firmin F, Crouch R (2009). Splinting versus casting of "torus" fractures to the distal radius in the paediatric patient presenting at the emergency department (ED): a literature review. Int Emerg Nurs.

[25] Williams KG, Smith G, Luhmann SJ, Mao J, Gunn JD 3rd, Luhmann JD (2013). A randomized controlled trial of cast versus splint for distal radial buckle fracture: an evaluation of satisfaction, convenience, and preference. Pediatr Emerg Care.

[26] Kennedy SA, Slobogean GP, Mulpuri K (2010). Does degree of immobilization influence refracture rate in the forearm buckle fracture?. J Pediatr Orthop B.

